# Preface to Special Topic: Invited Papers of the 3rd International Conference on Ultrafast Structural Dynamics

**DOI:** 10.1063/1.4947078

**Published:** 2016-04-25

**Authors:** S. L. Johnson

**Affiliations:** Institute for Quantum Electronics, Eidgenössische Technische Hochschule (ETH) Zurich, 8093 Zürich, Switzerland

## Abstract

The ability to visualize the real-time dynamics of atomic, magnetic, and electronic structure is widely recognized in many fields as a key element underpinning many important processes in chemistry, materials science, and biology. The need for an improved understanding of such processes becomes acute as energy conversion processes on fast time scales become increasingly relevant to problems in science and technology. This special issue, containing invited papers from participants at the 3rd International Conference on Ultrafast Structural Dynamics held June 10–12, 2015 in Zurich, Switzerland, discusses several recent developments in this area.

Over the last several years, there have been enormous advances in several different technical approaches to the problem of visualizing structural dynamics, both experimental and theoretical. On the experimental side, new developments in the creation of intense, femtosecond-duration short-wavelength radiation have led to several relevant capabilities.^1–7^ One of these is ultrafast diffraction, now applied widely using x-ray and electron based sources to study femtosecond dynamics in crystals.^8,9^ Similar methods can be applied to study short range order in disordered systems such as chemicals in solution.^10^ Ultrafast microscopy is another potential application of such sources that is finding increasing application.^11^

Ultrafast linear spectroscopies with wavelengths ranging from the visible to x-ray radiation are another technique that allows access to predominantly electronic structural dynamics. Here, developments in the brightness and tunability of femtosecond pulsed high energy photons have also pushed the frontier for progress in developing such applications.^12^ More generally, time-resolved linear spectroscopy is widely applicable to a variety of systems, ranging from chemicals in a solution^13,14^ to warm dense matter.^15^

Going beyond linear spectroscopy, multidimensional spectroscopy offers a way to study the flow of energy and its connection to structure in molecular systems.^16^ These methods are uniquely able to disentangle couplings between different parts of such systems, couplings which are often at the heart of understanding the energy landscape of excited materials. In the near future, new capabilities for coherent multi-color experiments in the VUV and x-ray range may offer very exciting new capabilities for extending the capabilities of multidimensional spectroscopy.^17^ This promises to enable the study of how charge is transported within a molecule on attosecond time scales, giving fundamental insights on the relationship of electronic structure to molecular function.^18^ The next years will be extremely exciting as experiments begin to explore this new frontier of structural dynamics.

A critical need for improved theory has also been widely recognized in the last few years, both on the level of direct experimental interpretation as well as a tool for understanding more general aspects of dynamics through models. This special issue reports on some of the recent works in this area.^19–21^ Further advances in this area will likely prove essential as new experimental techniques are able to reveal ever more subtle aspects of dynamics of electronic, magnetic, and atomic structures.
